# Study of the polymorphisms of cyclooxygenase-2 (−765G>C) and 5-lipoxygenase (1708G>A) in patients with colorectal cancer

**DOI:** 10.3892/ol.2013.1732

**Published:** 2013-12-05

**Authors:** CÉLIA APARECIDA MARQUES PIMENTA, FLAVIA ROCHE MOREIRA LATINI, JACQUELINE MIRANDA DE LIMA, TIAGO DONIZETTI DA SILVA, ALEDSON VITOR FELIPE, VANESSA MARIA DE LIMA PAZINE, NORA MANOUKIAN FORONES

**Affiliations:** Gastroenterology Division, Federal University of São Paulo, São Paulo 04023900, Brazil

**Keywords:** colorectal, cancer, polymorphism, cyclooxygenase, lipoxygenase

## Abstract

Colorectal cancer (CRC) is the fourth most common cause of cancer-related mortality worldwide. Genetic alterations have been associated with an increased risk of cancer and greater tumor aggressiveness. Cyclooxygenase-2 (COX-2) and 5-lipoxygenase (5-LOX) genes are important in cell cycle regulation, tumor growth and prostaglandin synthesis. The aim of the present study was to investigate the association between polymorphisms in the COX-2 and 5-LOX genes and the risk of CRC. A case-control study was conducted in patients with CRC matched for gender and age to a control group. DNA was extracted from peripheral leukocytes, and the polymorphisms were analyzed by polymerase chain reaction-restriction fragment length polymorphism and gene sequencing. A specific questionnaire was applied to evaluate smoking, excessive alcohol consumption, physical activity, non-steroidal anti-inflammatory drug use and meat, fiber and fat intake. A total of 185 patients with CRC and 146 controls were studied. The heterozygous GC genotype of the COX-2 gene polymorphism was the most common in the two groups (60.0% in CRC patients and 52.7% in controls). The CC genotype was associated with an increased risk of CRC (odds ratio, 3.63; 95% confidence interval, 1.31–10.1; P=0.013). The homozygous wild-type genotype of the 5-LOX gene polymorphism was detected in 72.4% of the CRC patients and in 71.2% of the control subjects. The homozygous mutant genotype (CC) of the COX-2 gene is an independent risk factor for CRC. No association was found between 5-LOX genotypes and CRC.

## Introduction

Colorectal cancer (CRC) is the third most common cancer and the fourth most frequent cause of cancer-related mortality worldwide. Colon cancer does not exhibit a gender preference, but rectal cancer is 20–50% more prevalent in males. Despite advances in early diagnosis and treatment, 30–40% of patients with CRC succumb to the disease (1). In Brazil, CRC is the fifth most frequent cause of mortality in males and the third in females (2).

A sequence of mutations in the genes involved in cell cycle control and apoptosis transform normal mucosa cells into malignant cells (3). Advances in molecular biology techniques have permitted an improved understanding of the genetic and molecular mechanisms involved in cancer development. Nevertheless, the initial event responsible for the malignant transformation of normal cells remains unclear. The gene transcriptional effect is inhibited by hypermethylation in the promoter region and oxidative damage to nuclear DNA, which are two of the main mechanisms associated with the early stages of colorectal carcinogenesis (4).

A single nucleotide polymorphism (SNP) is a genetic change in a nucleotide of the DNA sequence that occurs in >1% of the population (5). SNPs usually occur in non-coding regions, however, SNPs that occur in the coding regions of genes are frequently associated with various diseases, including cancer (5). Numerous genetic alterations, including polymorphisms in the cyclooxygenase (COX)-2 and 5-lipoxygenase (LOX) genes, have been associated with an increased risk of CRC and a poor prognosis, as well as a high fat intake, which increases the risk of developing tumors due to arachidonic acid metabolism that produces proinflammatory substances (6). There are three main enzymes involved in the metabolism of arachidonic acid, the COXs, the LOXs and cytochrome P450 (7). These genes are involved in cell cycle regulation, tumor growth and prostaglandin production. COX catalyzes the transformation of arachidonic acid into endoperoxide, which is enzymatically converted into substances that are involved in platelet aggregation, blood vessel dilatation and chemotaxis (8).

There are three isoforms of COX (COX-1, -2 and -3), and COX-2 has been shown to play a role in the carcinogenesis of colon, breast, prostate and esophageal cancer (?). In 2002, the COX-2 gene -765G>C polymorphism was described by Papafili *et al* and associated with a higher susceptibility to cancer (9). Resistance to apoptosis mediated by COX-2 is a central mechanism of tumorigenesis (10).

LOX, described in 1976 (11), is an additional enzyme involved in the transformation of arachidonic acid into prostaglandins and prostacyclins, and has been associated with a risk of CRC. Studies investigating isoforms of LOX that play a role in the control mechanisms of cancer emergence and progression have increased over the past years (12). The synthesis of proinflammatory mediators, such as leukotrienes, are catalyzed by LOX. Leukotrienes stimulate colon cell proliferation and inhibit apoptosis (13).

The aim of the present study was to analyze the -765G>C polymorphism in the COX-2 gene and the -1708G>A polymorphism in the 5-LOX gene in patients with CRC, and to correlate these polymorphisms with lifestyle and dietary habits.

## Patients and methods

### Patients and lifestyle habits

In a prospective sequential study, patients with CRC were compared with a control group selected from subjects without cancer or gastrointestinal symptoms observed during routine examination at the Central Laboratory of the Federal University of São Paulo (São Paulo, Brazil). All patients were born in Brazil and were treated by the Oncology Group of the Paulista School of Medicine (São Paulo, Brazil) between March 2009 and December 2010. Colorectal adenocarcinoma of the colon or rectum was confirmed by the pathologist. The study was approved by the Ethics Committee of the Federal University of São Paulo and all patients signed an informed written consent form.

Patients and controls answered a questionnaire concerning lifestyle habits, including smoking status (non-smokers and current and former smokers), alcohol consumption and physical activity. The frequency of meat, fruit, vegetable and fat intake, and the use of non-steroidal anti-inflammatory drugs (NSAIDs), was also evaluated. The body mass index (BMI; kg/m^2^) was calculated and the subjects were classified as malnourished, well-nourished or overweight. Peripheral blood was collected for extraction of genomic DNA.

### Analysis of the COX-2 -765G>C polymorphism

Leukocyte DNA was extracted from peripheral venous blood collected with ethylenediaminetetraacetic acid using the Qiagen^®^ Spin Blood Mini kit (Qiagen, Hilden, Germany). The COX-2 gene polymorphism was investigated by polymerase chain reaction (PCR) and restriction fragment length polymorphism (RFLP) analysis. The results were confirmed by genotyping in an ABI Prism 3100 genetic analyzer (Applied Biosystems, Carlsbad, CA, USA). Genomic DNA was amplified using the following primers: Forward, 5′-ATTCTGGCCATCGCCGCTTC-3′ and reverse, 5′-CTCCTTGTTTCTTGGAAAGAGACG-3′.

Following amplification, the PCR products were digested with Acil (New England Biolabs, Ipswich, MA, USA). The digestion products were separated on 2% agarose gels stained with ethidium bromide and visualized under ultraviolet light. The GG, GC and CC genotypes were analyzed in the two groups.

### Analysis of the 5-LOX 1708G>A polymorphism

The following primers were used for amplification of the 5-LOX 1708G>A polymorphism: Forward, 5′-GCACTGTATAGCATGTAC ATTA-3′ and reverse, 5′-CGTGACCCATTTTGAGTTAG-3′. The PCR products were purified using the QIAquick PCR Purification kit (Qiagen) and sequenced in an ABI Prism 3100 genetic analyzer (Applied Biosystems). The Sequence Scanner v1.0 program (Applied Biosystems) was used for electropherogram analysis.

### Statistical analysis

Statistical analyses were performed using SPSS version 16.0 (SPSS Inc., Chicago, IL, USA). Student’s t-test was used to compare ages between the groups. Differences in the polymorphisms between the two groups were determined by the χ^2^ test. This test was also used to compare clinical and epidemiological variables between COX-2 and 5-LOX genotypes and alleles in the group of cancer patients. Odds ratios (ORs) and 95% confidence intervals (CIs) were calculated to evaluate the association between the risk of developing cancer and these variables. Multivariate logistic regression was performed to identify risk factors. P<0.05 was considered to indicate a statistically significant difference and a confidence interval of 95% were used.

## Results

### Patient analysis

A total of 185 patients with CRC and 146 control subjects were studied. The mean age of patients with CRC was 62.7 years (standard deviation=13.1) and there were 99 females. No difference in age, gender or BMI was observed between the groups. The frequency of smokers (17%) and subjects practicing physical activity (44%) was higher in the control group. No differences were identified in the consumption of alcohol (P=0.391), fruits (P=0.706), vegetables (P=0.577), cereals (P=0.935) or red meat (P=0.495) between the groups. By contrast, fat intake was higher in the cancer patients (P=0.05) (Table I).

### Genotype distribution

**Figure 1:**
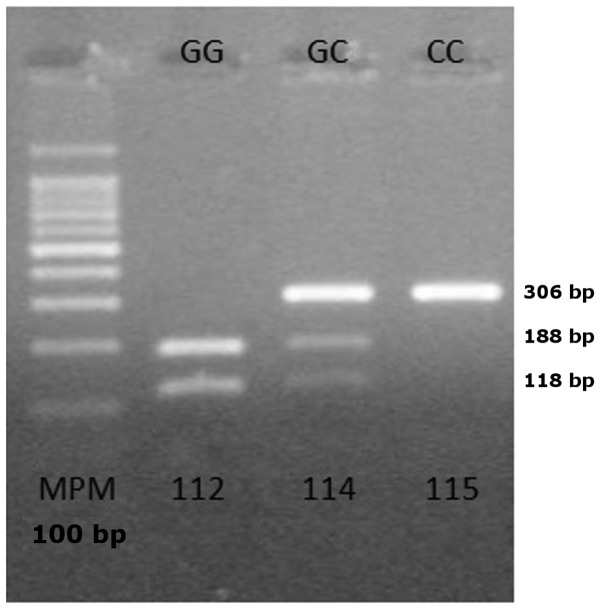
Polymerase chain reaction-restriction fragment length polymorphism (PCR-RFLP) for the study of COX-2. The wild-type genotype (GG) presents two fragments of 118 and 188 bp (patient 112). The heterozygous genotype (GC) presents three bands of 306, 188 and 118 bp (patient 114). The homozygous mutant genotype (CC) destroys the restriction site, and digestion produces a fragment of 306 bp (patient 115). COX-2, cyclooxygenase-2.

In the control group, the genotype distribution of the two polymorphisms was in accordance with the Hardy-Weinberg equilibrium (P>0.05). Analysis of the COX-2 polymorphism by PCR-RFLP revealed two bands of 118 and 188 bp in subjects carrying the homozygous wild-type genotype (GG), three bands of 306, 188 and 118 bp in subjects carrying the heterozygous genotype (GC) and one band of 306 bp in subjects with the homozygous mutant genotype (CC) (Fig. 1).

**Figure 2:**
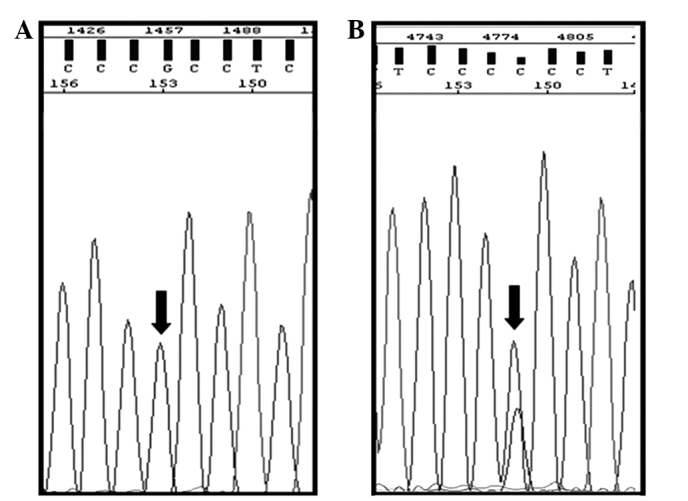
Electropherogram polymorphism printout of cyclooxygenase-2 (COX-2) from an automated sequencer. Sequencing reactions of colorectal cancer showed (A) wild-type GG (patient 217) and (B) heterozygous GC (patient 214) highlighted regions where polymorphisms were observed.

The heterozygous GC genotype was the most common in the two groups. The CC genotype was more frequent in the cancer group (P=0.013) and was associated with an increased risk of CRC (OR, 2.20; 95% CI, 1.02–4.76). Allele C was also associated with a higher risk of cancer (Table II). The PCR-RFLP results of COX-2 were confirmed in specific samples by sequencing (Fig. 2).

**Figure 3:**
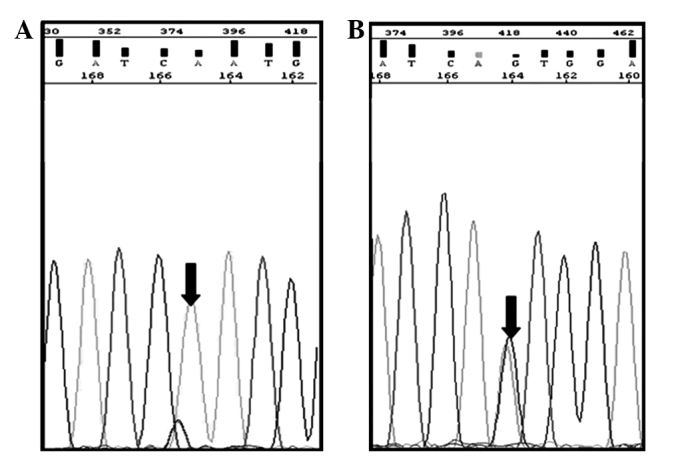
Electropherogram polymorphism printout of 5-lipoxygenase (5-LOX) from an automated sequencer. Sequencing reactions of colorectal cancer showed (A) mutant homozygous AA (patient 3) and (B) heterozygous GA (patient 43) highlighted regions where polymorphisms were observed.

With regard to the 5-LOX polymorphism (Fig. 3), 72.4% of patients with CRC and 71.2% of control subjects were homozygous for the wild-type. No difference was observed between groups (Table II).

### Statistical analysis

Multivariate analysis showed an increased risk of cancer in subjects who did not practice physical exercise or who consumed fat ≥3 times/week. This analysis confirmed the CC genotype as an independent risk factor for developing cancer (Table III).

## Discussion

The present study investigated whether polymorphisms of COX-2 and 5-LOX are associated with the risk of CRC, in addition to the correlation between these polymorphisms and lifestyle.

A high intake of fats causes changes in the intestinal flora that increase the concentration of bile acids, cell proliferation and prostaglandin production and thus, the inflammatory process (14). The consumption of red meat has been associated with an increased production of free radicals, which cause oxidative damage to epithelial cells and genetic mutations (15). The patients of the current study reported a high frequency of fat intake and a low frequency of physical activity.

It is known that a sedentary lifestyle has been associated with an increased cancer risk, and low levels of COX-2 expression have been observed in rats submitted to physical exercise (16).

In the present study, the use of NSAIDs was more common among the control subjects, possibly due to the high prevalence of patients with cardiovascular disease in this group. Previous epidemiological studies have shown that acetylsalicylic acid is able to reduce the incidence of CRC by 40–50%. Among the mechanisms of action of NSAIDs, four inhibitors of the receptor activation of peroxisome γ into cellular DNA may be cited, inactivating the genes responsible for development, metabolism, cell growth and differentiation (17). Notably, in the present study, smokers were more common in the control group, but no statistically significant correlation was identified between smoking and the risk of CRC. This may be explained by the method of collecting the patient history, which depended on the questioning of patients on their smoking habits and not the smoking index rate. Previously, Pandey *et al*(18) found no correlation between smoking and COX-2 polymorphisms in patients with cancer of the cervix.

Although certain studies have previously reported an association between alcohol, low vitamin intake and cancer, no consensus exists in the literature (19). In the present study, no correlation was identified between alcohol consumption and cancer risk.

COX-2 polymorphisms have been associated with an increased risk of cancer. Among the 10 COX-2 polymorphisms that have been associated with cancer risk, −765G>C and −1195A>G are the most common in CRC (20,21). The COX-2 gene −765G>C polymorphism has been shown to modify the gene transcription that may cause an alteration in the binding capacity of specificity protein 1 and consequently, an increased expression of COX-2. The homozygous mutant CC genotype has been associated with a high incidence of leukemia (22) and bladder (23), breast (24) and esophageal cancer (25). In the present study, the risk of CRC was higher among subjects carrying the CC genotype even after control for the other variables studied. In addition, Biramijamal *et al* found a correlation between this polymorphism and an increased risk of CRC (OR, 7.1; 95% CI, 1.03–5.26) (26). By contrast, a decreased risk of developing hepatocarcinoma was observed in patients with the CC genotype (27). A higher risk of CRC and gastric cancer (28) has been described for subjects carrying the wild-type GG genotype, but this association has not been confirmed by others. Kristinsson *et al,* studying COX-2 polymorphisms (765G>C and 1195A>G), observed that the GG/GG haplotype was more frequent in esophageal adenocarcinoma (29). No association of these polymorphisms was observed in the oral and pharyngeal cancer risk (30).

In the current study, the mutant CC genotype of the COX-2 gene polymorphism increased the risk of CRC by ~2-fold (Table II) and by ~5-fold when combined with physical inactivity and consumption of fried foods.

Racial and ethnic differences between the populations studied may explain these contradictory results since the distribution of COX-2 polymorphisms differs considerably between the populations.

The polymorphism in the 5-LOX gene, resulting in a change of guanine to adenine at position 1,708, has been studied in CRC (31) and lung, kidney, bladder, pancreas, esophageal and prostate cancer. 5-LOX is an enzyme that is involved in the control of cell proliferation and apoptosis inhibition, and has been associated with CRC risk. Previously, Jiang *et al*(32) observed that the levels of 12- and 5-LOX were particularly high in the tumors of patients who succumbed to breast cancer. In CRC (33) and pancreatic cancer (6), 5-LOX expression studied by immunohistochemistry and RT-PCR was found to correlate with tumor expansion and invasion of blood vessels.

Wang *et al*(34) previously reported an increased risk of breast cancer in females with a high intake of n-6 polyunsaturated fats and the 1700G>A polymorphism in the 5-LOX gene. In addition, Shen *et al*(35) described an increased risk of lung cancer in the sputum of patients carrying the 12-LOX (GA) polymorphism.

Although no association was identified between CRC and the 5-LOX polymorphism in the present study, patients with genotypes GA/AA exhibited a 2.5-fold higher chance of developing cancer when they consumed meat >3 times per week. These genotypes (GA/AA) were also associated with an increased CRC risk in patients with a high intake of fats upon univariate analysis.

In conclusion, the current study identified a significant difference in the distribution of the COX-2 gene −765CC polymorphism between patients with CRC and controls. The presence of the −765CC genotype was associated with an increased risk of CRC. The CC genotype mutated COX-2 increased the risk of CRC by ~2 times and was not associated with physical activity. In addition, the intake of fried food increased the risk of CRC by 5 times. However, future studies are required to investigate the possible association of this polymorphism with the prognosis of CRC.

## Figures and Tables

**Table I:** Characteristics of patients and OR for CRC according to fat intake, physical activity and alcohol consumption.

Variable	Cases, n (%)	Controls, n (%)	P-value^a^	OR^b^ (95% CI)	P-value^a^	OR^c^ (95% CI)
Gender						
Male	86 (46.5)	71 (48.6)	0.698			
Female	99 (53.5)	75 (51.4)				
Age, years						
Male	62.0±12.7	61.2±15.6	0.357^d^			
Female	63.4±13.4	60.8±15.9	0.433^d^			
Total	62.7±13.1	61.0±15.8	0.543^d^			
Fat intake^e^						
Low	10 (5.4)	12 (8.2)		1.00 Reference		1.00 Reference
Medium	90 (48.7)	97 (66.4)	0.699	1.23 (0.42–3.60)	0.812	1.11 (0.46–2.70)
High	85 (45.9)	37 (25.4)	0.050	3.11 (1.00–9.69)	0.031	2.76 (1.09–6.64)
Physical activity						
Yes	34 (18.4)	66 (45.2)		1.00 Reference		1.00 Reference
No	151 (81.6)	80 (54.8)	<0.001	3.83 (2.08–7.06)	<0.001	3.66 (2.23–6.01)
NSAIDs use						
Yes	1 (0.5)	44 (30.1)		1.00 Reference		1.00 Reference
No	184 (99.5)	102 (69.9)	<0.001	203 (17.8–2327)	<0.001	79.4 (10.8–584)
Alcohol drinker						
No	134 (72.4)	103 (70.6)		1.00 Reference		1.00 Reference
Yes	51 (27.6)	43 (29.4)	0.706	0.47 (0.40–1.38)	0.706	0.91 (0.56–1.47)
Cereals						
Yes	87 (47.1)	68 (46.6)		1.00 Reference		1.00 Reference
No	98 (52.9)	78 (53.4)	0.916	0.97( 0.57–1.67)	0.935	0.98 (0.64–1.52)
Fruits^e^						
High	110 (59.4)	83 (56.9)		1.00 Reference		1.00 Reference
Medium	69 (37.2)	61 (41.8)	0.610	0.89 (0.56–1.41)	0.487	0.85 (0.55–1.33)
Low	6 (3.4)	2 (1.4)	0.178	3.13 (0.60–16.4)	0.325	2.26 (0.45–11.5)
Vegetables^e^						
High	104 (56.2)	80 (54.8)		1.00 Reference		1.00 Reference
Medium	69 (37.3)	61 (41.8)	0.811	0.93 (0.54–1.62)	0.546	0.87 (0.55–1.37)
Low	12 (6.5)	5 (3.4)	0.095	3.33 (0.81–13.7)	0.267	1.85 (0.62–5.45)
Cancer site						
Colon	103 (55.3)					
Rectum	83 (44.7)					
Cancer stage						
I	28 (15.1)					
II	84 (45.4)					
III	52 (28.1)					
IV	21 (11.4)					

a Pearson χ^2^ test;

b OR and CI adjusted by age and gender;

c unadjusted OR and CI;

d two-sample t-test;

e low, 1 time/week;

medium, 2–3-times/week; and high, >3 times/week. Data are presented as the mean ± standard deviation for continuous variables and n (%) for categorical variables. OR, odds ratio; CRC, colorectal cancer; CI, confidence interval; NSAIDs, non-steroidal anti-inflammatory drugs.

**Table II:** 5-LOX and COX-2 genotypes of patients and OR for CCR.

Genotype	n (%)	n (%)	P-value	OR^a^ (95% CI)	P-value	OR^b^ (95% CI)
5-LOX	185	146				
G/G	134 (72.4)	104 (71.2)	0.723^c^	1.00 Reference	0.970^c^	1.00 Reference
G/A	46 (24.9)	38 (26.1)	0.229	0.68 (0.36–1.28)	0.807	0.94 (0.57–1.55)
A/A	5 (2.7)	4 (2.7)	0.940	0.94 (0.19–4.75)	0.965	0.97 (0.25–3.70)
COX-2						
G/G	49 (26.5)	56 (38.4)	0.004^c^	1.00 Reference	0.050^c^	1.00 Reference
G/C	111 (60.0)	77 (52.7)	0.015	2.11 (1.16–3.83)	0.042	1.65 (1.02–2.67)
C/C	25 (13.5)	13 (8.9)	0.013	3.63 (1.31–10.1)	0.046	2.20 (1.02–4.76)
G Allele	209 (65.5)	189 (70.5)		1.00 Reference		1.00 Reference
C Allele	161 (43.5)	103 (29.5)	<0.001	1.94 (1.14–3.31)	<0.05	1.41 (1.03–1.94)
G/C + C/C	136 (73.5)	90 (61.6)		1.00 Reference		1.00 Reference
G/G	49 (26.5)	56 (38.4)	0.006	0.44 (0.25–0.79)	0.021	0.58 (0.36–0.92)
G/C + G/G	160 (86.5)	133 (91.1)		1.00 Reference		1.00 Reference
C/C	25 (13.5)	13 (8.9)	0.093	2.21 (0.88–5.56)	0.191	1.60 (0.79–3.25)

a OR and CI adjusted by age and gender;

b unadjusted OR and CI;

c genotype trend.

5-LOX, 5-lipoxygenase; COX-2, cyclooxygenase-2; OR, odds ratio; CCR, colorectal cancer; CI, confidence interval.

**Table III:** Multivariate logistic regression analysis stratified by the selected variables.

Variable	Cases, n (%)	Controls (n=146), n (%)	P-value	OR^a^ (95% CI)
Fat intake^b^				
Low	10 (5.4)	12 (8.2)		1.00 Reference
Medium	90 (48.7)	97 (66.4)	0.952	1.04 (0.34–3.16)
High	85 (45.9)	37 (25.4)	0.149	2.40 (0.73–7.89)
Physical activity				
Yes	34 (18.4)	66 (45.2)		1.00 Reference
No	151 (81.6)	80 (54.8)	<0.001	3.90 (2.04–7.43)
COX-2 genotypes				
G/G	49 (26.5)	56 (38.4)		1.00 Reference
G/C	111 (60.0)	77 (52.7)	0.010	2.30 (1.21–4.34)
C/C	25 (13.5)	13 (8.9)	0.003	5.03 (1.74–14.6)

a OR and CI adjusted by age and gender;

b low, 1 time/week;

medium, 2–3- times/week; and high, >3 times/week. OR, odds ratio; CI, confidence interval; COX-2, cyclooxygenase-2.
